# Biomechanical and histomorphometric characterization of the melatonin treatment effect in the carotid artery subjected to hypobaric hypoxia

**DOI:** 10.3389/fbioe.2025.1554004

**Published:** 2025-04-16

**Authors:** Eugenio Rivera, Alvaro Navarrete, Claudio M. Garcia-Herrera, Leonardo Gordillo, Enrique Cerda, Diego J. Celentano, Alejandro Gonzalez-Candia, Emilio A. Herrera

**Affiliations:** ^1^ Departamento de Ingeniería Mecánica, Universidad de Santiago de Chile, Santiago, Chile; ^2^ Departamento de Física, Universidad de Santiago de Chile, Santiago, Chile; ^3^ Department of Mechanical and Metallurgical Engineering, Pontificia Universidad Católica de Chile, Santiago, Chile; ^4^ Institute of Health Sciences, Universidad de O’Higgins, Rancagua, Chile; ^5^ Pathophysiology Program, Faculty of Medicine, Institute of Biomedical Sciences (ICBM), Universidad de Chile, Santiago, Chile; ^6^ International Center for Andean Studies (INCAS), Universidad de Chile, Santiago, Chile

**Keywords:** biomechanics, carotid artery, hypobaric hypoxia, melatonin, biomechanical tests, finite element method

## Abstract

This study aims to assess the efficacy of melatonin in mitigating the adverse effects of hypobaric hypoxia on the cardiovascular system of neonatal lambs (30 days old). Two groups were considered for this purpose: (i) Melatonin-treated group (N = 5) and (ii) Control group (N = 6) without treatment. All subjects were exposed to hypobaric hypoxia during gestation and perinatal periods, with melatonin administered after birth. The study focused on the carotid artery, a known predictor of cardiovascular risk. Biomechanical tests, morphometric, and histological measurements were conducted, and a numerical model was developed based on the biomechanical data. Key findings showed remodeling effects: Firstly, a realignment of collagen fibers towards a longitudinal direction was observed with melatonin treatment, similar to non-hypoxic arteries. Second, changes in residual stress and *ex-vivo* luminal radius were noted, aiming to reduce wall stress and increase vascular resistance. These changes indicate an antihypertensive response, reducing the effects of increased blood pressure and flow due to hypobaric hypoxia. This study demonstrates that biomechanical and histomorphometric methodologies effectively assess the beneficial effects of melatonin treatment under hypobaric hypoxia exposure.

## 1 Introduction

Vasculature is particularly affected by changes in the physiological environment, manifested in adaptative/maladaptative processes (Sehgal et al., 2019). Accordingly, biomechanics has been proven as a successful tool in assessing vascular impairments, particularly related to the aging process (Haskett et al., 2010), along with the development and progression of cardiovascular diseases (Lasheras, 2007; Walsh et al., 2014; Murtada et al., 2021; Tong et al., 2023).

Focusing attention on the arteries, their characteristic microstructural configuration plays a key role in the biomechanical response under physiological conditions. The mechanical behavior is mainly governed by the action of elastin and collagen fibers (extracellular matrix (ECM)), along with the smooth muscle cells (SMC) (Pukaluk et al., 2024; Holzapfel and Ogden, 2018). Kochova et al., 2012 (Kochová et al., 2012) found out the passive biomechanical effect of each one of these components by its respective degradation, through the inflation-deflation testing. They discovered that elastin is related to an increased diameter at physiological pressure levels without determining stiffness changes, which is attributed to collagen influence. In contrast, SMC content induces both diameter change and arterial wall stiffening at supra-physiological pressures.

Hypoxia, characterized by low oxygen levels at the cellular and tissue levels, is detrimental to maintaining normal physiological processes (Ream et al., 2008). In the gestational stage, hypoxia induced by a high-altitude environment (commonly referred to as hypobaric hypoxia, HH) is classified as a kind of preplacental hypoxia, due to both mother and fetus being subjected to this condition (Hutter et al., 2010). Besides the geographic conditions, fetal hypoxia can also develop as a result of biological abnormalities, namely, impaired development of the placenta during the early pregnancy period (Eskild et al., 2016), umbilical cord occlusion (Kawagoe et al., 1999), and maternal diabetes (Klemetti et al., 2021). In particular, this condition triggers an alteration in normal development in the perinatal period, where chronic exposure to HH during pregnancy has revealed high incidence of intrauterine growth restriction (IUGR) (Parraguez et al., 2005; brown and GiussanI, 2024). IUGR has been closely related to cardiopulmonary complications during life (Rueda-Clausen et al., 2009), including premature pulmonary hypertension (Herrera et al., 2010; Papamatheakis et al., 2013; Ding et al., 2020; Sigaeva et al., 2019; Steinhorn, 2017) and reduced cardiac performance (Patterson and Zhang, 2010) which leads in turn, to higher risks of adult cardiovascular disease (Giussani and Davidge, 2013; Ream et al., 2008).

Aiming to mitigate the pulmonary hypertension effects, the performance of vasodilator treatments has been assessed. In the physiological context, melatonin is naturally released by the pineal gland to the body at night, aiming to regulate the circadian and seasonal rhythms (Xu et al., 2018; Nelson and Drazen, 2000; Zisapel, 2018), along with pubertal development (Pandi-Perumal et al., 2008; Olcese, 2020). Different research has shown that when used as a treatment, melatonin exhibits antioxidant properties (Figueroa et al., 2021). This is particularly crucial in different types of hypoxia exposure conditions (Debevec et al., 2017; Farías et al., 2012; González-Candia et al., 2019). In addition, this drug has successfully mitigated effects linked to pulmonary arterial hypertension (Torres et al., 2015; Maarman and Lecour, 2021; Hung et al., 2017; Yildiz and Balcioğlu, 2024; Astorga et al., 2018). Simultaneously, several alternative treatments have been proposed for these same goals, i.e., atrial natriuretic peptide (Wiedemann et al., 2001; Werner et al., 2016; Hussain et al., 2019), cinaciguat (Beñaldo et al., 2022; Chester et al., 2011; Laubrie et al., 2023), and allopurinol (Liu-Shiu-Cheong et al., 2020; Gokcen et al., 2022).

Physiologically, the common carotid artery (CCA) plays a crucial role in the cardiovascular system, by perfusing oxygenated blood from the heart to the brain territory (Sethi et al., 2023). Structurally, it is classified as a conductive or elastic-type artery, denoted by a higher content of elastic fibers than smooth muscle cells, unlike in the peripheral muscular arteries (Brown et al., 2018). This distinctive feature impacts a high level of vascular compliance, essential for responding to the significant blood pressure fluctuations this kind of artery is subjected to (Peace et al., 2018). Different authors have shown the impact of intrauterine growth restriction (IUGR) on the carotid arteries of various animal models along perinatal development. Kucukbas and Doğan (2023) evaluated hemodynamics parameters (i.e., pulsatility and resistance indexes, along with peak systolic velocity) via ultrasound Doppler technique in the common carotid artery of fetuses with IUGR, determining an abnormal blood flow under this condition to the difference of those subjected to normal pregnancy. Paz et al. (2019) assessed the changes in carotid morphology under IUGR conditions, specifically noting a reduction in luminal diameter, while observing no alterations in vascular reactivity, neither in contractile nor dilation *ex-vivo* function. Cañas et al., (2018) determined the passive mechanical properties and residual stress quantification via ring tensile and ring opening tests in the aorta, carotid, and femoral arteries of guinea pigs fetuses, in pregnancies subjected to progressive uterine artery occlusion. The main results did not find conclusive evidence about changes in the mechanical behavior and residual stress in carotid arteries. Dodson et al. (2013a) studied the effect of placental insufficiency-induced IUGR in umbilical and carotid arteries of sheep near-term fetuses, determining a decrement in compliance for both studied arteries, measured through the inflation-extension test. In addition, histological observations reveal arterial remodeling, denoted by an increment in the content of elastic and collagen fibers in the carotid.

Focusing on the biomechanical aspects of perinatal development under chronic HH-induced IUGR, there is limited research (Rivera et al., 2020; Rivera et al., 2021; Navarrete et al., 2024), and even less on those of the carotid artery (Navarrete et al., 2020). On the other hand, there is little information about the drug-based treatment effects. Melatonin has been found to have a vasodilator effect in pulmonary circulation, but its impact on systemic circulation arteries is yet to be established. Based on the fact that there is evidence of alteration in the normal characteristics of the carotid artery regarding biomechanics and morphological parameters, under the mentioned condition, we hypothesize that administration of melatonin treatment in newborn lambs exposed to chronic HH during both pregnancy and postnatal periods alter the carotid artery mechanical response and its morphology.

To verify the research hypothesis, a numerical-experimental study of preclinical nature was carried out in the common carotid artery of newborn lambs gestated and bred in high altitude conditions. The passive mechanical properties were determined through biomechanical tests (explained in Section 2.2), and the corresponding characterization was performed using a suitable constitutive model (detailed in Section 2.3.1). Once the model has been characterized, a well-established numerical simulation procedure (detailed in Section 2.3.2) determines the residual stress field in the arterial wall. In addition, histological and ultrastructural assessments were conducted to determine the microstructure and morphology of the artery wall (Section 3.3).

## 2 Materials and methods

### 2.1 Materials

The study was conducted on the carotid artery of lambs whose conception, gestation, and neonatal period took place in an environment of hypobaric hypoxia at the International Center for Andean Studies (INCAS) of Universidad de Chile, located in the town of Putre at 3,600 m above sea level (m.a.s.l.).

The number of animals required for the study was determined following the 3 R s of good practices in animal experimentation (Replacement, Reduction, and Refinement of animals, from “The principles of human experimental techniques”, 1959). The samples were divided into two experimental groups.

• Control group (CN), consisting of six specimens without pharmacological treatment.
• Melatonin group (MN), consisting of five specimens treated with melatonin medication.

The pharmacological treatment consists of the oral administration of 1 mg/kg of melatonin at approximately 8 p.m. for 20 days, between four and 23 days of age. After delivery, neonates are left without interventions for 2–3 days to ensure maternal-newborn attachment and proper recognition of their mothers, which ensures good lactation. It has been done with the purpose of increasing melatonin levels at a steady rate without altering the circadian rhythm of the lambs. Euthanasia occurs at 30 days of age, through the intravenous administration of 100 mg/kg of sodium thiopental. Subsequently, the dissection and extraction of the carotid artery were carried out, and within a period not exceeding 24 h, the corresponding biomechanical tests were performed. All animal procedures for this study were carried out with the approval of the Bioethics Committee on animal research of the Faculty of Medicine of Universidad de Chile (CBA #0761 FMUCH).

### 2.2 Experimental methods

#### 2.2.1 Biomechanical tests

Different biomechanical tests were conducted in the carotid artery. They were perfomed inmedialty after disection procedure. During the entire process (both extraction and testing), the sample was immersed in physiological saline solution. Moreover, after extraction, the arterial segment was preserved at 4 ° C. During testing, the tissue was gradually brought to normal body temperature (39 $\pm 1^{{}^{\circ}}$ C), following the protocol established by Rivera et al. (2020).

##### 2.2.1.1 Uniaxial tensile test

**FIGURE 1:**
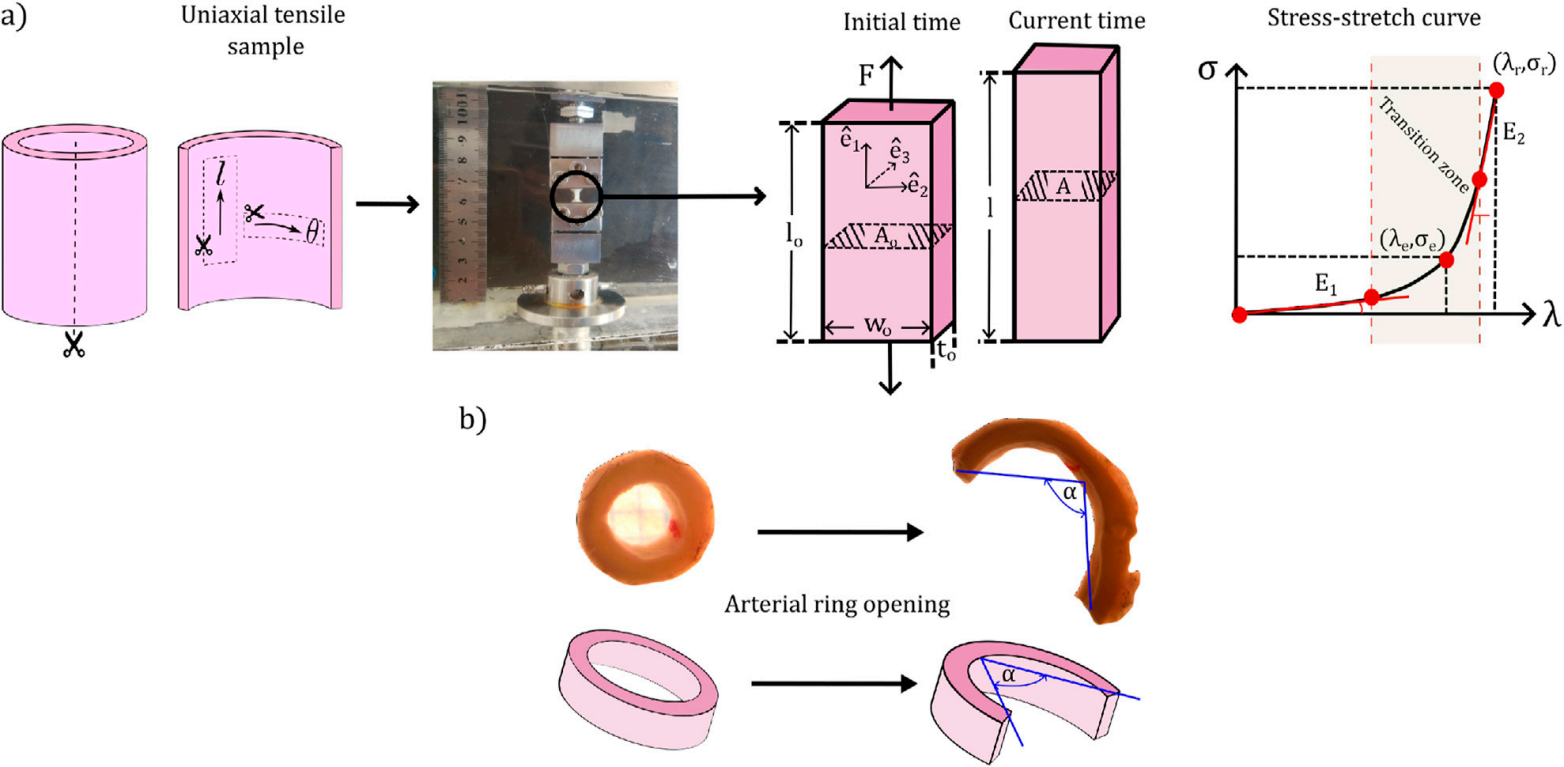
Set-up of biomechanical tests and representative measurements. **(a)** Uniaxial tensile test. **(b)** Ring opening test.

Figure 1a, schematizes the main aspects of the experimental procedure involved in the uniaxial tensile test. For each sample, two uniaxial tensile tests were performed: one in longitudinal and the other in circumferential directions (denoted by the directions l and θ , respectively, according to the Figure 1a). Initially, a tubular section of the arterial sample, approximately 2.5 cm long, was opened through a longitudinal cut. Then, rectangular-shaped samples were extracted in both directions considered. Each sample was mounted in the testing machine Instron 3342 (equipped with a 10 N load cell with a precision ±0.1 N). Before the test begins, the initial dimensions were measured, where both the length $\left(l_{0}\right)$ and width $\left(w_{0}\right)$ were obtained via image processing using the software ImageJ (Schneider et al., 2012). These measurements were performed from an image of the mounted sample placed beside a ruler (see Figure 1a), whose error is ± 1 mm. On the other hand, the thickness $t_{0}$ was determined via histological images, according to the refered below in Section 2.2.2).

The test was conducted by stretching the sample in the direction ${\hat{e}}_{1}$ of Figure 1a, at a quasi-static speed of 1.5 mm/min. From the instantaneous force (F) measured by the load cell, and the actual length (l) of the sample, the Cauchy stress was calculated through the expression $\left(\sigma_{1}=\frac{F}{A}\right)$ , where A is the instantaneous cross-sectional area of the sample. The stretch $\lambda_{1}$ during the test was calculated as the ratio between the actual and the initial length of the sample $\left(\lambda_{1}=\frac{l}{l_{0}}\right)$ . Alternatively, whereas the sample is stretched in direction ${\hat{e}}_{1}$ , the other two directions ( ${\hat{e}}_{2}$ , ${\hat{e}}_{3}$ shown in Figure 1a) are shortened, and the stretch is denoted as $\lambda_{2}$ and $\lambda_{3}$ , respectively. As the tissue was assumed to be incompressible (Rivera et al., 2020), the instantaneous cross-sectional area can be determined as $\left(A=\frac{A_{0}}{\lambda_{1}}\right)$ , where $\left(A_{0}=w_{0}\;t_{0}\right)$ is the initial one.

From the experimental dates, stress-stretch curves were generated, which consider the dataset of force from the beginning of the test, until when it reaches its maximum value (right side of the Figure 1a). From them, the following characteristic parameters were quantified: the slope at low strain levels $\left(E_{1}\right)$ and at high strain levels $\left(E_{2}\right)$ , the coordinates of the elbow point $\left(\lambda_{e},\sigma_{e}\right)$ , and the rupture point $\left(\lambda_{r},\sigma_{r}\right)$ . In specific, the slopes $E_{1}$ and $E_{2}$ were obtained by a linear fitting of the data, considering the highest quantity of points until the coefficient of determination $r^{2}$ be equal to 0.95 (Rivera et al., 2020). According to the referential stress-stretch curve from Figure 1, the elbow is defined as the midpoint of the transition zone, which delimits the low and high stretch levels. Its value is determined by the mean stress-stretch coordinate between the stretch on the curve defining the end of the slope for $E_{1}$ and the beginning of the slope for $E_{2}$ . Finally, the rupture point is taken as the last stress-stretch coordinate value considered. (Rivera et al., 2020; García-Herrera et al., 2012).

##### 2.2.1.2 Ring opening test

**FIGURE 2:**
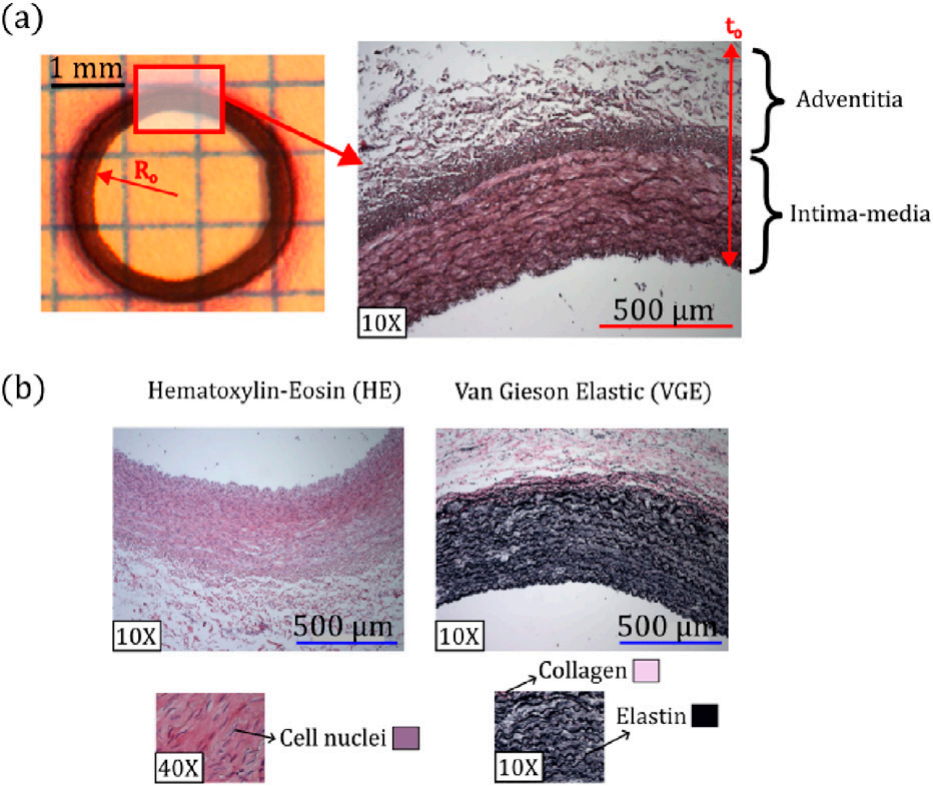
**(a)** Representative parameters of morphometric measurements. **(b)** Representative histological images of Hematoxylin-Eosin (HE) and van Gieson Elastic (VGE) staining, along with colors and zones where elastic fibers, collagen, and cell nuclei were quantified.

In this research, a 4-mm arterial ring segment was extracted from the carotid artery, which was radially cut to measure its opening angle α . The transversal section of the ring as characterized by the lumninal radius $R_{0}$ along with the wall thickness $t_{0}$ , both measured through histological images, using the software ImageJ , such as is exhibited in Figure 2a. The measurements were performed 20 min after the cut procedure to avoid any transient effect (Rivera et al., 2020). Figure 1b schematizes the experimental process of this test. The opening angle was retrieved by image processing using the software ImageJ.

#### 2.2.2 Histomorphometry

Figure 2a schematizes the protocol to obtain the morphometric measurements of samples, corresponding to the external radius $R_{\text{o}}$ and arterial wall thickness $t_{\text{o}}$ . $R_{\text{o}}$ was measured through images of the arterial wall cross-section, which were captured by magnifying glass Motic SMZ-161 (left image of Figure 2a), while $t_{\text{o}}$ was determined from 10X histological images (right image of Figure 2a), which includes both the intima-media and adventitia layers.

To determine the microstructural composition, a well-known histological procedure was applied to the artery under study (Carson and Hladik, 2009). In particular, two stains were used for this purpose (see Figure 2b). On the one hand, the Hematoxylin-Eosin (HE) staining was used to identify the cell nuclei (visualized with purple color), determining the cell nuclei density (number of cells per unit surface). Each cell nucleus was identified through a color thresholding process. The overall number of nuclei per image, was then quantified using a particle analyzer, which counts these regions based on a minimum size of 50 ${\text{pixels}}^{2}$ (value determined by inspection). On the other hand, the Van Gieson Elastic (VGE) staining quantifies two extracellular matrix components: elastic fibers (identified by black color) and collagen fibers (pink color). Specifically, to determine the percentages corresponding to collagen and elastic fibers, the histological images undergo a color thresholding. This process defines three parameters: hue, saturation, and brightness, which are adjusted to identify the characteristic colors of the fibers. All these processes of counting, described below, have been developed using the software ImageJ.

#### 2.2.3 Statistical procedure

All data of the samples were expressed as mean ± standard error of the mean (SEM), calculated as the ratio of the standard deviation to the square root of the number of specimens. The representation of the mean population differences was quantified through the 95% confidence interval (CI), along with the p−value of a suitable statistical test. To this end, a well-defined procedure was used to determine the statistical test to be used (Navarrete et al., 2024). First, the Shapiro-Wilk test was used to assess the normality of the data sets. When both samples are normally distributed, the F-test was used to compare the variances between the groups. Based on these two results, the appropriate statistical method was chosen to assess differences between means. The unpaired t-test was used when both groups followed a normal distribution and equal variances, Welch’s t-test was also considered in groups with normal distribution, but different population variance; while a non-parametric Mann-Whitney U-test was used when the distribution of one or both groups was not normal. Differences were considered significant when p−value≤0.05 . The software GraphPad Prism 6.01 (GraphPad Software Inc., San Diego, CA, USA) was used for this purpose.

### 2.3 Numerical methods

#### 2.3.1 Constitutive modelling

Continuum mechanics models soft tissue’s purely elastic mechanical response as those of hyperelastic materials (Dwivedi et al., 2022). According to the intrinsic characteristics of the material under study, a suitable hyperelastic constitutive model must be considered for this purpose. In particular, several authors have considered the artery wall to be an isotropic ground matrix fiber-reinforced (Huh et al., 2019; Lee et al., 2019; Pagoulatou et al., 2021). This idea is supported by observations of its microstructure, where the isotropic matrix represents the non-preferential direction of the elastic fibers, whereas the fibrous part in the model reflects the preferential orientation of the collagen fibers along the arterial wall. Thus, we opted for the transversely isotropic Gasser-Holzapfel-Ogden (GHO) model in this study (Gasser et al., 2006).

In general, for hyperelastic materials, the stress-strain relationship is determined based on the strain energy function (W) , which for the GHO model is defined as follows:

$$W\left(C\right)=\frac{\mu }{2}\;\left(I_{1}-3\right)+\frac{k_{1}}{2\;k_{2}}\;\underset{\text{i}=4,6}{\sum }\left[exp\left(k_{2}\;{\left[\kappa \;I_{1}+\left(1-3\kappa \right)\;I_{\text{i}}-1\right]}^{2}\right)-1\right],$$ (1)

which involves the following terms: (i) The first invariant of the right Cauchy-Green strain tensor (C) , denoted as $\left(I_{1}=tr\left(C\right)\right)$ . (ii) Two pseudo-invariants of this strain tensor, $I_{4}={\hat{a}}_{1}\cdot C\;\;{\hat{a}}_{1}$ and $I_{6}={\hat{a}}_{2}\cdot C\;\;{\hat{a}}_{2}$ , both related to the orientation of the two-symmetrical family of fibers in the plane defined by the circumferential and longitudinal axes, each one represented by ${\hat{a}}_{1}=\left[sin\left(\gamma \right),cos\left(\gamma \right),0\right]$ and ${\hat{a}}_{2}=\left[-sin\left(\gamma \right),cos\left(\gamma \right),0\right]$ , where γ is defined as the mean angle of each family of fibers measured from longitudinal direction of the arterial duct. (iii) The set of material constants μ , $k_{1}$ , $k_{2}$ and κ , where the first three are referred to stiffness both in isotropic matrix ground and fibers, meanwhile the last one to the degree of dispersion of both fiber families considered (0≤κ≤1/3) .

A particular consideration about arterial tissue is its incompressibility, where the volume does not change in the face of any characteristic deformation state (García-Herrera et al., 2016). In this case, the definition of the stress state is not only deformation-dependent, since added hydrostatic pressure p on the material is unable to deform it. The stress-strain relationship, specifically for incompressible materials, is stated through the Lagrangian multiplier approach, and it can be deducted from the following expression:

$$\sigma =2J^{-1}F\frac{\partial W\left(\bar{C}\right)}{\partial C}F^{\text{T}}-pI,$$ (2)

where σ is the Cauchy stress tensor, $\bar{C}=J^{-2/3}\;C$ the isochoric part of the tensor C with $J=det\left(F\right)$ (equal to one under incompressibility condition), p the Lagrange multiplier that represents the already mentioned hydrostatic pressure (related to volumetric component), and I is the identity tensor. Equations 1, 2 yield the stress-strain relationship as stated by:

$$\sigma =J^{-1}FSF^{\text{T}}-pI,$$ (3)

where S is the Second Piola Kircchoff stress tensor, whose specific expression for the GHO model is:

$$\begin{array}{cc} S_{ij} & =\mu I_{\text{ij}}+2\kappa k_{1}\left[\kappa I_{1}+\left(1-3\kappa \right)I_{4}-1\right]{exp}^{{\text{k}}_{2}{\left[\kappa I_{1}+\left(1-3\kappa \right)I_{4}-1\right]}^{2}}I_{\text{ij}}+\\ & \;2\kappa k_{1}\left[\kappa I_{1}+\left(1-3\kappa \right)I_{6}-1\right]{exp}^{{\text{k}}_{2}{\left[\kappa I_{1}+\left(1-3\kappa \right)I_{6}-1\right]}^{2}}I_{\text{ij}}+\\ & \;2k_{1}\left(1-3\kappa \right)\left[\kappa I_{1}+\left(1-3\kappa \right)I_{4}-1\right]{exp}^{{\text{k}}_{2}{\left[\kappa I_{1}+\left(1-3\kappa \right)I_{4}-1\right]}^{2}}\varrho_{\text{ij}}+\\ & \;2k_{1}\left(1-3\kappa \right)\left[\kappa I_{1}+\left(1-3\kappa \right)I_{6}-1\right]{exp}^{{\text{k}}_{2}{\left[\kappa I_{1}+\left(1-3\kappa \right)I_{6}-1\right]}^{2}}\varrho_{\text{ij}}-pC_{\text{ij}}^{-1}, \end{array}$$ (4)

where the tensor $\varrho_{ij}$ is defined as:

$$\varrho_{\text{ij}}=\left\{\begin{array}{c} {cos}^{2}\gamma ,if\;i=j=1\;\\ {sin}^{2}\gamma ,if\;i=j=2\;\\ 0,if\;i\neq j\wedge i,j=1,2\; \end{array}\right.$$ (5)

Particular case: uniaxial tensile test.

Three-dimensional spatial region designated for the tensile test (Figure 1a) is defined by the orthogonal unit vector triad $\left({\hat{e}}_{1},{\hat{e}}_{2},{\hat{e}}_{3}\right)$ . The sub index ${□}_{1}$ denotes the direction where deformation is applied (either longitudinal and circumferential, according to the two directions tested), whereas ${□}_{2}$ and ${□}_{3}$ are the other two directions (being ${□}_{3}$ the radial direction in all cases).

In particular, in the case of uniaxial tensile test, the definition of right Cauchy-Green strain tensor corresponds to the diagonal matrix $C=diag\left[\lambda_{1}^{2},\lambda_{2}^{2},\lambda_{3}^{2}\right]$ , and the Cauchy stress tensor is $\sigma =diag\left[\sigma_{1},0,0\right]$ . Moreover, the incompressibility condition implies that $\lambda_{3}=1/\lambda_{1}\lambda_{2}$ . Through the Equations 3–5 and the previously referred particularizations of the uniaxial tensile test, the semi-analytical expressions for the GHO model can be deduced. Further details about these equations are exhibited in the study of García-Herrera et al. (2016).

The set of material parameters defined by the GHO model (i.e., μ , $k_{1}$ , $k_{2}$ , κ , γ ) were determined via numerical fitting, by considering the experimental information provided by the uniaxial tensile test both in longitudinal and circumferential directions. To solve the parameter determination, the gradient-based optimization Levenberg-Marquardt algorithm was used to address the non-linear least square problem (Gundiah et al., 2009). The coefficient of determination for each direction considered ( $r_{\theta }^{2}$ and $r_{\text{l}}^{2}$ ) is the metric used to evaluate the fitting quality, widely used in biomechanical studies (Saw et al., 2018; Avril et al., 2013; Guo et al., 2023).

#### 2.3.2 Numerical simulation of the ring closure

Aiming to determine the residual stress field across the artery wall, the numerical simulation of the inverse process to the experimental ring opening test is described below. Experimental information of the ring opening angle α (Section 2.2.1) along with the initial *ex-vivo* geometry of the arterial ring ( $R_{\text{o}}$ and $t_{\text{o}}$ , Section 3.3), are required as input data (Cañas et al., 2018).

**FIGURE 3:**
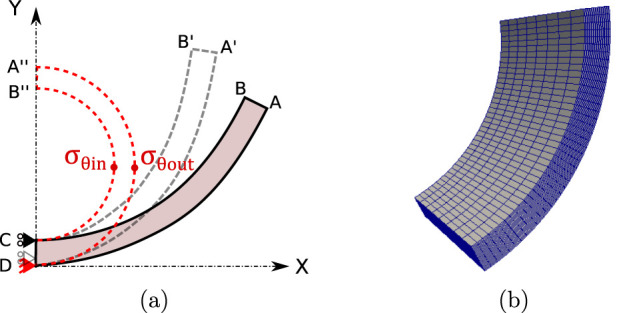
**(a)** Representation 2D of the boundary conditions for the computational simulation of the closure of the rings, **(b)** 3D finite element mesh [generated in Gmsh (Geuzaine and Remacle, 2009) and visualized in ParaView software].

Figure 3a, outlines the numerical procedure in the simulation, where material homogeneity and geometrical symmetry were considered (García-Herrera et al., 2016). The referential configuration (region ABCD) corresponds to a stress-free, open and stabilized arterial ring. The numerical ring-closure was achieved by imposing displacements on the line AB, which moves via A’B’ and finishes in the closed ring configuration (A″B″ position).

To achieve the adequate reconstruction of the closed geometry, the mean perimeter of the closed arterial ring is related to the arc formed by its respective open configuration. An analytical expression (Equation 6) is used to relate the mean radius of the open configuration $R_{\alpha }$ with the mean radius of the closed one $R_{\text{m}}$ ,

$$R_{\alpha }=\frac{\pi }{\pi -\alpha }\cdot R_{m},$$ (6)

where $R_{m}=R_{\text{o}}+\frac{t_{\text{o}}}{2}$ , and α is in radians.

Simulations were performed using the Finite Element Method (FEM) via an *in-house* code (Celentano et al., 1996; Celentano, 2001). In the FEM context, incompressibility in hyperelastic models (as GHO) was implemented through a nearly incompressible condition, by adding an appropriate penalty parameter k that represents the bulk modulus, which is higher than the shear modulus parameter μ to enforce the incompressibility $\left(k\approx 10^{3}\mu \right)$ (Yosibash and Priel, 2011). Figure 3b exhibits the 3D mesh used, which is composed of 8,000 hexahedra elements and 9,594 nodes.

From simulation, two characteristic values were considered: circumferential residual Cauchy stress in the inner $\sigma_{\theta in}$ and outer radius $\sigma_{\theta out}$ , such as is exhibited in Figure 3a.

## 3 Results

From the experimental and numerical procedure described in Section 2, the biomechanical, structural, and morphometric results are listed in detail below.

### 3.1 Uniaxial tensile test

Table 1 exhibits the mechanical parameters directly determined from tensile curves for both study groups, as displayed in the procedure stated in Section 2.2.1.

**TABLE 1:** Average ± SEM, along with mean difference and 95% confidence interval of biomechanical parameters derived from the stress-strain curve for the carotid artery: Slopes to low $\left(E_{1}\right)$ and high $\left(E_{2}\right)$ deformation levels, elbow coordinates ( $\lambda_{\text{e}}$ , $\sigma_{\text{e}}$ ) and rupture coordinates $\left(\lambda_{\text{r}},\sigma_{\text{r}}\right)$ .

	CN-l	95% CI	MN-l	95% CI
$E_{1}\;\left[kPa\right]$	81.9 ± 15.5	$\left[42.0;121.7\right]$	128.2 ± 42.8	$\left[9.3;247.2\right]$
$E_{2}\;\left[kPa\right]$	1894 ± 289	$\left[1150;2637\right]$	2355 ± 404	$\left[1069;3642\right]$
$\lambda_{\text{e}}\;\left[\frac{mm}{mm}\right]$	1.72 ± 0.07	$\left[1.54;1.90\right]$	1.66 ± 0.06	$\left[1.50;1.81\right]$
$\sigma_{\text{e}}\;\left[kPa\right]$	119.5 ± 12.9	$\left[86.2;152.7\right]$	127.8 ± 21.9	$\left[67.1;188.5\right]$
$\lambda_{\text{r}}\;\left[\frac{mm}{mm}\right]$	2.73 ± 0.14	$\left[2.34;3.12\right]$	2.59 ± 0.05	$\left[2.45;2.73\right]$
$\sigma_{\text{r}}\;\left[kPa\right]$	1,648 ± 305	$\left[864.1;2432\right]$	1,676 ± 185	$\left[1163;2189\right]$

	CN- θ	95% CI	MN- θ	95% CI
$E_{1}\;\left[kPa\right]$	206.1 ± 82.9	$\left[-57.8;469.9\right]$	138.3 ± 47.3	$\left[6.91;269.7\right]$
$E_{2}\;\left[kPa\right]$	2005 ± 486	$\left[655.8;3354\right]$	1935 ± 246	$\left[1152;2718\right]$
$\lambda_{\text{e}}\;\left[\frac{mm}{mm}\right]$	1.53 ± 0.06	$\left[1.38;1.70\right]$	1.61 ± 0.09	$\left[1.37;1.85\right]$
$\sigma_{\text{e}}\;\left[kPa\right]$	188.7 ± 54.2	$\left[38.2;339.3\right]$	154.3 ± 22.9	$\left[90.6;218.0\right]$
$\lambda_{\text{r}}\;\left[\frac{mm}{mm}\right]$	2.25 ± 0.10	$\left[1.96;2.53\right]$	2.31 ± 0.15	$\left[1.89;2.72\right]$
$\sigma_{\text{r}}\;\left[kPa\right]$	1,230 ± 354	$\left[247.0;2212\right]$	1,382 ± 334	$\left[455.4;2309\right]$

From the Table 1, material stiffening at low and high deformation levels, ( $E_{1}$ and $E_{2}$ , respectively), shows different trends in the longitudinal and circumferential directions when both experimental groups were contrasted. Particularly, the mean stiffness in the longitudinal direction of the blood vessel at low and high deformation levels, increases under melatonin treatment (l- $E_{1}$ : CN ⇒ 81.9 ± 15.5 kPa, MN ⇒ 128.2 ± 42.8 kPa, p-value = 0.30), (l- $E_{2}$ : CN ⇒ 1894 ± 289 kPa, MN ⇒ 2355 ± 404 kPa, p-value = 0.37). Otherwise, mean values of $E_{1}$ and $E_{2}$ decrease in the circumferential direction in the treated group ( θ - $E_{1}$ : CN ⇒ 206.1 ± 82.9 kPa, MN ⇒ 138.3 ± 47.3 kPa, p-value = 0.48) and ( θ - $E_{2}$ : CN ⇒ 2005 ± 486 kPa, MN ⇒ 1935 ± 246 kPa, p-value = 0.91), respectively. The transition between low and high deformation levels in stress-stretch curves of the uniaxial tensile test, as represented by $\lambda_{\text{e}}$ and $\sigma_{\text{e}}$ . Both parameters behave differently in the directions considered, where the mean elbow stretch in the longitudinal one experiences a diminution (l- $\lambda_{\text{e}}$ : CN ⇒ 1.72 ± 0.07, MN ⇒ 1.66 ± 0.06, p-value = 0.50), while the circumferential one increases ( θ - $\lambda_{\text{e}}$ : CN ⇒ 1.53 ± 0.06, MN ⇒ 1.61 ± 0.09, p-value = 0.48). Conversely, whereas the stress level increases along the longitudinal direction (l- $\sigma_{\text{e}}$ : CN ⇒ 119.5 ± 12.9 kPa, MN ⇒ 127.8 ± 21.9 kPa, p-value > 1.00), the same value for the circumferential one decreases under melatonin treatment ( θ - $\sigma_{\text{e}}$ : CN ⇒ 188.7 ± 54.2 kPa, MN ⇒ 154.3 ± 22.9 kPa, p-value = 0.57). The rupture zone, quantified by its corresponding stretch $\left(\lambda_{\text{r}}\right)$ and stress $\left(\sigma_{\text{r}}\right)$ values, responds differently in each direction. Specifically, for the longitudinal axis, the mean rupture stretch level decreased in the melatonin-treated group (l- $\lambda_{\text{r}}$ : CN ⇒ 2.73 ± 0.14, MN ⇒ 2.59 ± 0.05, p-value = 0.39); meanwhile, for the circumferential direction, it increased ( θ - $\lambda_{\text{r}}$ : CN ⇒ 2.25 ± 0.10, MN ⇒ 2.31 ± 0.15, p-value = 0.75). Concerning the mean values of rupture stress, they exhibit the same characteristic for both directions, showing an increment under melatonin (l- $\sigma_{\text{r}}$ : CN ⇒ 1648 ± 305 kPa, MN ⇒ 1676 ± 185 kPa, p-value = 0.94), ( θ - $\sigma_{\text{r}}$ : CN ⇒ 1230 ± 354 kPa, MN ⇒ 1382 ± 334 kPa, p-value = 0.76).

According to the fitting procedure carried out for the hyperelastic model (GHO) in this study (Section 2.3.1), the corresponding material parameters have been displayed in Table 2.

**TABLE 2:** Values for the GHO model parameters were optimized to fit the experimental data obtained from uniaxial tension tests. The coefficients $r_{\theta }^{2}$ and $r_{\text{l}}^{2}$ facilitate the adjustment of the model in the circumferential and longitudinal orientations, respectively.

Group	μ [kPa]	$k_{1}$ [kPa]	$k_{2}$	κ	γ [°]	$r_{\theta }^{2}$	$r_{\text{l}}^{2}$
Control, CN	35.0	73.0	0.07	0.27	74.5	0.981	0.990
Melatonin, MN	33.0	79.0	0.06	0.26	52.5	0.992	0.992

When comparing the GHO parameters, fitted from the mean stress-stretch curves obtained from control and melatonin groups, μ , related to the material stiffness of the isotropic part, they exhibit similar values between them (difference of 6%). In the same way, most of the fibrous-like parameters do not show major differences, e.g., $k_{1}$ , $k_{2}$ and κ in none of the cases differ above 15% . However, the collagen fiber orientation, represented by γ , exhibits noticeable changes (close to 30%), diminishing its value from 74.5 ° to 52.5 ° .

**FIGURE 4:**
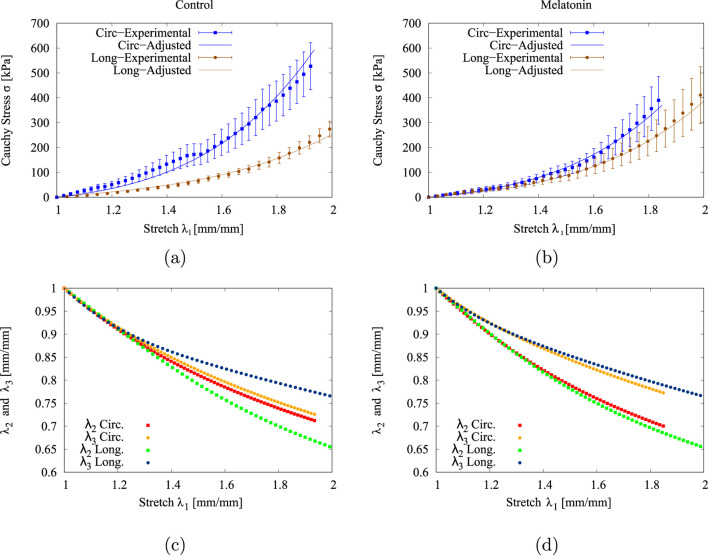
Experimental ( ± SEM) and fitted stress-strain curves from the uniaxial tensile test for **(a)** control (CN) and **(b)** melatonin (MN) groups. Physical consistency of the GHO parameters obtained, represented through the stretch values along the width and thickness of the tensile sample, for **(c)** control (CN) and **(d)** melatonin (MN) groups.

Figures 4a, b, depict the experimental ( ± SEM) and numerical stress-stretch curves for the control and treated groups. The goodness of fitting, as represented by the r-square values in the two directions $r_{\theta }^{2}$ and $r_{\text{l}}^{2}$ . Table 2) displays these values, which were close to 0.99 in all cases, which reflects a good fitting quality. On the other hand, Figures 4c, d display the numerical stretch values on the two orthogonal axes (width $\lambda_{2}$ and thickness $\lambda_{3}$ ) regarding on which the stretching $\lambda_{1}$ was applied. The decay of all curves as the stretch increases ensures the physical consistency of the fittings performed.

### 3.2 Ring-opening test and residual stress

Following the procedure described in Section 2.2.1, the opening angle α in the control group was 111±15° with 95% CI $\left[71.4;149.8\right]$ , whereas its value corresponds to 124±16° with 95% CI $\left[80.7;167.3\right]$ in the case of the melatonin-treated group, without significant mean differences between groups (p-value = 0.56).

The mean α value, along with $R_{\text{o}}$ and $t_{\text{o}}$ were used in the computational reconstruction of the open arterial ring for both groups of study. From the numerical simulation, followed under the procedure detailed in Section 2.3.2, three representative values were obtained: the circumferential Cauchy stress both in the inner radius zone $\left(\sigma_{\theta_{\text{in}}}\right)$ and outer radius zone $\left(\sigma_{\theta_{\text{out}}}\right)$ and the absolute difference between both values $\Delta \sigma_{\theta }$ . All these values were schematized and displayed in Table 3.

**TABLE 3:** Circumferential residual stress in inner and outer arterial radius ( $\sigma_{\theta_{in}}$ and $\sigma_{\theta_{\mathrm{out}}}$ ), along with the absolute difference between them $\left(\Delta \sigma_{\theta }\right)$ . The scheme displays the specific location from where they have been obtained.

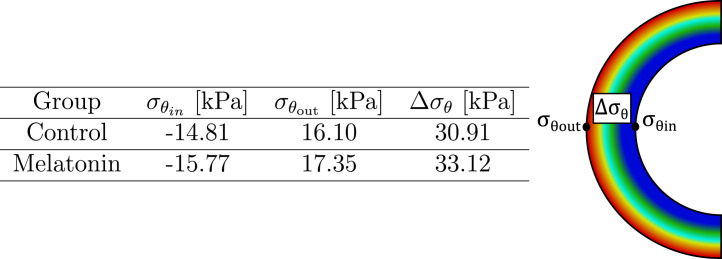

### 3.3 Histomorphometry

The different characteristics that describe the morphometric and microstructural composition of the carotid artery wall are detailed in Table 4.

**TABLE 4:** Average ± SEM, along with mean difference and 95% confidence interval of morphometric and histological results for both groups of study.

	Control (CN)	95% CI	Melatonin (MN)	95% CI
Morphometry				
Luminal radius $R_{\text{o}}$ (mm)	1.28 ± 0.07	$\left[1.10;1.46\right]$	1.14 ± 0.04	$\left[1.02;1.25\right]$
Thickness $t_{\text{o}}$ (mm)	0.53 ± 0.04	$\left[0.44;0.62\right]$	0.48 ± 0.06	$\left[0.30;0.66\right]$
Intima-Media (%)	52.7 ± 2.7	$\left[45.8;59.6\right]$	54.7 ± 2.1	$\left[48.9;60.5\right]$
Adventitia (%)	47.3 ± 6.2	$\left[31.4;63.2\right]$	45.4 ± 6.3	$\left[27.9;62.9\right]$
Histology (media layer)				
Elastic fibers (%)	31.3 ± 3.7	$\left[21.0;41.5\right]$	39.6 ± 3.2	$\left[29.4;49.8\right]$
Collagen (%)	7.0 ± 0.9	$\left[4.5;9.5\right]$	6.8 ± 0.1	$\left[6.4;7.2\right]$
Cell nuclei density (cells/ ${mm}^{2}$ )	2524 ± 66	$\left[2340;2708\right]$	2570 ± 142	$\left[2119;3021\right]$

Within structural measures, luminal radius $\left(R_{\text{o}}\right)$ , overall thickness $\left(t_{\text{o}}\right)$ and percentage of thickness comprising both the intima-media and adventitia layers were obtained for both groups. Although the arteries of animals treated with melatonin have lower values in $R_{\text{o}}$ and $t_{\text{o}}$ , these differences are not significant ( $R_{\text{o}}$ : CN ⇒ 1.28 ± 0.07 mm, MN ⇒ 1.14 ± 0.04 mm, p-value = 0.12) and ( $t_{\text{o}}$ : CN ⇒ 0.53 ± 0.04 mm, MN ⇒ 0.48 ± 0.06 mm, p-value = 0.50).

In the same way, changes in both mean values exhibited below are related to an increase in the wall percentage covered by the intima-media (CN ⇒ 52.7 ± 2.7%, MN ⇒ 54.7 ± 2.1%, p-value = 0.59). In turn, the percentage of adventitia decreases in the MN group regarding the control (CN ⇒ 47.3 ± 6.2%, MN ⇒ 45.4 ± 6.3%, p-value = 0.84).

Histological results, which include the percentage of collagen and elastic fibers, along with the density of the cell nuclei (Table 4), were performed following the protocol stated in Section 2.2. Elastic fibers exhibit a tendency of higher content in arteries treated with melatonin (CN ⇒ 31.3 ± 3.7%, MN ⇒ 39.6 ± 3.2%, p-value = 0.29). Meanwhile, the percentage of collagen fibers was similar for both groups (CN ⇒ 7.0 ± 0.9%, MN ⇒ 6.8 ± 0.1%, p-value = 0.19) and cell nuclei density also lack of changes (CN ⇒ 2524 ± 66 cells/ ${mm}^{2}$ , MN ⇒ 2571 ± 142 cells/ ${mm}^{2}$ , p-value = 0.76).

## 4 Discussion

This preclinical study has been conducted to identify the main aspects related to HH exposure and the effects of drug-based treatment on biomechanics, morphometric and histology measurements.

Physiologically, the study on the carotid artery is of clinical relevance because alteration of its properties and structure has been widely identified as an indicator of cardiovascular risks, triggering adverse consequences mainly on the cerebral territory (Moghadasi et al., 2024; Hirata et al., 2006). Morphological, changes in intima-media and adventitia thickness have been assessed as indicators of atherosclerosis and coronary heart disease (Mohamed et al., 2023; Skilton et al., 2011; Ebrahimi, 2009). Furthermore, alteration of the microstructural components of the arterial wall, such as elastin degradation (Fonck et al., 2007) and density decrement (Kamenskiy et al., 2015), have been directly associated with atherosclerosis and hypertension. Likewise, the assessment of biomechanical metrics, such as 2D strain, stress, energy storage, and arterial stiffness, among others, have been linked to both cardiovascular complications (Kim et al., 2012; Lanne et al., 1994; Forsblad-D’Elia et al., 2021; Olver et al., 2016) and aging (Gkousioudi et al., 2022; Sherman et al., 2022). Therefore, the biomechanical, morphological, and structural characteristics assessed in the current work are relevant to determine potential adverse cardiovascular effects in the face of hypobaric hypoxia exposure.

From Table 1, no biomechanical parameter obtained exhibits any statistical differences between groups. However, the results obtained via numerical analysis, expressed in terms of the material parameters of the GHO hyperelastic model, show that the phenomenological parameter γ exhibits the most significant variations when comparing the numerical results in the two experimental groups, as shown in Table 2. This angular value shows a remarkable decrement for the melatonin-treated group [ ≈ 30% of percentage difference), which is interpreted as a collagen fiber reorientation towards the longitudinal direction compared to the control non-treated group. Interestingly, the previous study of Dodson al. (2013b)] coincides with this fact, which considers both the same arterial territory and experimental animal used in our research, has determined experimentally an alteration in the phenomenological parameter γ for near-term ewe fetuses under IUGR condition, which arises from the collagen fibers realignment such as has been observed via the second harmonic generation technique. In fact, in our results for newborn lambs treated with melatonin, the γ parameter returns to similar values to those obtained in the control group of the referred research (without IUGR condition), meanwhile in the study of Dodson al. (2013b), the fetuses under IUGR condition exhibit fiber alignment preferentially in the circumferential direction, in the same manner as has been determined in our findings (in hypoxic group). Therefore, these results suggest that melatonin reverses the effects of the HH condition, restoring features observed in non-hypoxic arteries.It is worth noting that, as the GHO model parameters were fitted from the average curves of the tensile tests, the numerical results reference potential alterations in the biomechanics resulting from the treatment. Further evidence can be given by considering the numerical fit for each specimen individually.

Residual stress plays a key role in arteries, which has been closely related to blood vessel remodeling (Cheng and Zhang, 2019). Biomechanically, the effect of residual stress arises intending to diminish the peaks or gradients in pressure on the artery wall, which is generated from blood pressure (Zahn and Balzani, 2018; Cañas et al., 2018). The residual stress field obtained from the numerical simulation procedure detailed in Section 2.3 is represented through the parameters shown in Table 3, whose values are dependent on the circumferential residual strain (quantified by the opening angle), and the material parameters from GHO model. In this sense, according to Equation 1, the first term of the GHO model (μ) can be interpreted as the parameter that describes the biomechanical effect at the physiological level, which is similar in both experimental groups (CN ⇒35kPa ; MN ⇒33kPa ). Likewise, from Section 3.2, the mean opening angle (α) was higher in the treated group (CN ⇒111° ; MN ⇒124° ). Table 3 confirms the previous findings, since both the inner $\sigma_{\theta_{\text{in}}}$ and outer arterial $\sigma_{\theta_{\text{out}}}$ regions have higher absolute values in compression and tension values for the melatonin group. As a consequence, the difference between the compressive and tensile values $\Delta \sigma_{\theta }$ in the melatonin group was also higher. These findings suggest that the increase in residual stress levels observed in groups treated with melatonin may contribute to reducing and balancing differences in the arterial wall stress to potential elevations in blood pressure associated with hypoxia exposure (Navarrete et al., 2024). Considering that residual stress is influenced by elastic fibers (Cardamone et al., 2009; Greenwald et al., 1997), the increment in the mean value of the opening angle α seems to be linked to a potential rise in the mean percentage of elastic fiber content (Table 4). However, there are no conclusive statistics to support this relationship.

Within the scope of this study, the effects of melatonin were examined only under HH conditions. The impact of the treatment at sea-level conditions was not addressed in this work. Precisely, this aspect would be interesting to address in future studies to evaluate the baseline parameters that the melatonin group exposed to HH should reach. In addition, an increase in the number of specimens in each group could lead to more conclusive results on the outcomes.

## 5 Conclusion

Through a coupled experimental-numerical approach, the influence of melatonin treatment on the biomechanical response in carotid arteries subjected to hypoxia hypobaric condition has been assessed. Aiming to this goal, several relevant biomechanical parameters have been determined for this goal, i.e., material stiffness, stress-stretch rupture levels, and residual stress. In addition, morphometric and histological measurements arise as further insights to give explanation to the mechanism that governs the characteristic mechanical response on the artery.

Melatonin treatment in animals exposed to gestational and perinatal hypobaric hypoxia primarily induces collagen fiber reorientation, rather than changes in extracellular matrix proliferation, degradation, or cell nuclei density. This is supported by biomechanical responses from uniaxial tensile tests and alterations in the hyperelastic model parameter γ linked to collagen fiber orientation. Previous studies confirm similar findings, showing melatonin tends to restore conditions observed in control groups. Additional changes, including reduced wall stress and increased vascular resistance, suggest an antihypertensive response related to cardiovascular pressure and blood flow regulation.

Future studies should focus on investigating the impact of different melatonin doses in distinct developmental stages, including long-term effects. New biomechanical analysis can be conducted on the artery wall, determining the effect of the active response, viscoelastic effects, and damage phenomenon, among others. Furthermore, the exploration of alternative biomechanical models encompassing growth and remodeling phenomena, as well as the impact of fiber degradation (Humphrey, 2021) can be explored. Moreover, novel ultrastructural techniques can be explored to obtain additional information regarding the microstructural phenomena carried out during the remodeling process on the arterial wall (i.e., second harmonic generation, atomic force microscopy, electron microscopy, and multiphoton imaging), which cannot be obtained through conventional histological techniques.

## Data Availability

The raw data supporting the conclusions of this article will be made available by the authors, without undue reservation.

## References

[B1] Astorga C. R. González-Candia A. Candia A. A. Figueroa E. G. Cañas D. Ebensperger G. (2018). Melatonin decreases pulmonary vascular remodeling and oxygen sensitivity in pulmonary hypertensive newborn lambs. Front. physiology 9, 185. 10.3389/FPHYS.2018.00185 PMC5845624 29559926

[B2] Avril S. Badel P. Gabr M. Sutton M. A. Lessner S. M. (2013). Biomechanics of porcine renal arteries and role of axial stretch. J. Biomechanical Eng. 135, 0810071. issn 01480731. Available from. 10.1115/1.4024685 PMC3705980 23722353

[B3] Beñaldo F. A. Araya-Quijada C. Ebensperger G. Herrera E. A. Reyes R. V. Moraga F. A. (2022). Cinaciguat (BAY-582667) modifies cardiopulmonary and systemic circulation in chronically hypoxic and pulmonary hypertensive neonatal lambs in the alto andino. Front. Physiology 13, 1. issn 1664042X. Available from. 10.3389/fphys.2022.864010 PMC9207417 35733986

[B4] Brown E. R. Giussani D. A. (2024). Cause of fetal growth restriction during high-altitude pregnancy. iScience 27, 109702. Available from. 10.1016/J.ISCI.2024.109702 38694168 PMC11061758

[B5] Brown I. A. M. Diederich L. Good M. E. DeLalio L. J. Murphy S. A. Cortese-Krott M. M. (2018). Vascular smooth muscle remodeling in conductive and resistance arteries in hypertension. Arteriosclerosis, Thrombosis, Vasc. Biol. 38, 1969–1985. issn 15244636. Available from. 10.1161/ATVBAHA.118.311229 PMC6205219 30354262

[B6] Cañas D. García-Herrera C. M. Herrera E. A. Celentano D. J. Krause B. J. (2018). Mechanical characterization of arteries affected by fetal growth restriction in Guinea pigs (*Cavia porcellus*). J. Mech. Behav. Biomed. Mater. 88, 92–101. issn 18780180. Available from. 10.1016/j.jmbbm.2018.08.010 30142566

[B7] Cardamone L. Valentín A. Eberth J. F. Humphrey J. D. (2009). Origin of axial prestretch and residual stress in arteries. Biomechanics Model. Mechanobiol. 8 (6), 431–446. Available from. 10.1007/S10237-008-0146-X PMC2891240 19123012

[B8] Carson F. Hladik C. (2009). *Histotechnology: a self-instructional*. 3ed. Am. Soc. Clin. Pathology.

[B9] Celentano D. (2001). A large strain thermoviscoplastic formulation for the solidification of S.G. cast iron in a green sand mould. Int. J. Plasticity 17, 1623–1658. Available from. 10.1016/S0749-6419(00)00095-4

[B10] Celentano D. Oller S. Oñate E. (1996). A coupled thermomechanical model for the solidification of cast metals. Int. J. Solids Struct. 33 (5), 647–673. Available from. 10.1016/0020-7683(95)00056-G

[B11] Cheng J. Zhang L. T. (2019). Simulation of vessel tissue remodeling with residual stress: an application to in-stent restenosis. Int. J. Smart Nano Mater. 10, 11–27. issn 1947542X. Available from. 10.1080/19475411.2018.1529002

[B12] Chester M. Seedorf G. Tourneux P. Gien J. Tseng N. Grover T. (2011). Cinaciguat, a soluble guanylate cyclase activator, augments cGMP after oxidative stress and causes pulmonary vasodilation in neonatal pulmonary hypertension. Am. J. Physiology - Lung Cell. Mol. Physiology 301, L755–L764. 10.1152/AJPLUNG.00138.2010 PMC3213988 21856817

[B13] Debevec T. Millet G. P. Pialoux V. (2017). Hypoxia-induced oxidative stress modulation with physical activity. Front. Physiology 8, 84. issn 1664042X. Available from. 10.3389/FPHYS.2017.00084 PMC5303750 28243207

[B14] Ding H. Luo Y. Hu K. Huang H. Liu P. Xiong M. (2020). Hypoxia *in utero* increases the risk of pulmonary hypertension in rat offspring and is associated with vasopressin type-2 receptor upregulation. Mol. Med. Rep. 22, 4173–4182. issn 17913004. Available from. 10.3892/MMR.2020.11533 33000260 PMC7533485

[B15] Dodson R. B. Rozance P. J. Fleenor B. S. Petrash C. C. Shoemaker L. G. Hunter K. S. (2013a). Increased arterial stiffness and extracellular matrix reorganization in intrauterine growth-restricted fetal sheep. Pediatr. Res. 73, 147–154. 10.1038/PR.2012.156 23154756 PMC3742323

[B16] Dodson R. B. Rozance P. J. Reina-Romo E. Ferguson V. L. Hunter K. S. (2013b). Hyperelastic remodeling in the intrauterine growth restricted (IUGR) carotid artery in the near-term fetus. J. biomechanics 46, 956–963. issn 00219290. Available from. 10.1016/J.JBIOMECH.2012.12.013 PMC3742322 23332229

[B17] Dwivedi K.Kr Lakhani P. Kumar S. Kumar N. (2022). A hyperelastic model to capture the mechanical behaviour and histological aspects of the soft tissues. J. Mech. Behav. Biomed. Mater. 126, 105013. Available from. 10.1016/j.jmbbm.2021.105013 34920323

[B18] Ebrahimi A. P. (2009). Mechanical properties of normal and diseased cerebrovascular system. J. Vasc. Interventional Neurology 2, 155–162. 10.5281/zenodo.10319619 PMC3317338 22518247

[B19] Eskild A. Strøm-Roum Marie E. Haavaldsen C. (2016). Does the biological response to fetal hypoxia involve angiogenesis, placental enlargement and preeclampsia? Paediatr. Perinat. Epidemiol. 30, 305–309. issn 13653016. Available from. 10.1111/PPE.12283 27038011 PMC4825407

[B20] Farías J. G. Zepeda A. B. Calaf G. M. (2012). Melatonin protects the heart, lungs and kidneys from oxidative stress under intermittent hypobaric hypoxia in rats. Biol. Res. 45, 81–85. issn 0716-9760. Available from. 10.4067/S0716-97602012000100011 22688988

[B21] Figueroa E. G. Gonzaléz-Candia A. Villanueva C. A. Ebensperger G. Reyes R. V. Llanos A. J. (2021). Beneficial effects of melatonin on prostanoids pathways in pulmonary hypertensive neonates. Vasc. Pharmacol. 138, 106853. issn 1537-1891. Available from. 10.1016/J.VPH.2021.106853 33766627

[B22] Fonck E. Prod'hom G. Roy S. Augsburger L. Rüfenacht D. A. Stergiopulos N. (2007). Effect of elastin degradation on carotid wall mechanics as assessed by a constituent-based biomechanical model. Am. J. Physiology - Heart Circulatory Physiology 292, H2754–H2763. issn 03636135. Available from. 10.1152/ajpheart.01108.2006 17237244

[B23] Forsblad-D’Elia H. Law L. Bengtsson K. Smeds J. Ketonen M. Sundström B. (2021). Biomechanical properties of common carotid arteries assessed by circumferential 2D strain and *β* stiffness index in patients with ankylosing spondylitis. J. rheumatology 48, 352–360. issn 1499-2752. Available from. 10.3899/JRHEUM.200146 32611672

[B24] García-Herrera C. M. Bustos C. A. Celentano D. J. Ortega R. (2016). Mechanical analysis of the ring opening test applied to human ascending aortas. vol. 5842, no. May. Available from 10.1080/10255842.2016.1183125 27178265

[B25] García-Herrera C. Atienza J. M. Rojo F. J. Claes E. Guinea G. V. Celentano D. J. (2012). Mechanical behaviour and rupture of normal and pathological human ascending aortic wall. Med. Biol. Eng. Comput. 50 (6), 559–566. 10.1007/s11517-012-0876-x 22391945

[B26] Gasser T. Christian O Ray W. Holzapfel G. A. (2006). Hyperelastic modelling of arterial layers with distributed collagen fibre orientations. J. R. Soc. Interface 3 (6), 15–35. issn 17425662. Available from. 10.1098/rsif.2005.0073 16849214 PMC1618483

[B27] Geuzaine C. Remacle J. F. (2009). Gmsh: a 3-D finite element mesh generator with built-in pre- and post-processing facilities. Int. J. Numer. Methods Eng. 79, 1309–1331. issn 00295981. Available from. 10.1002/NME.2579

[B28] Giussani D. A. Davidge S. T. (2013). Developmental programming of cardiovascular disease by prenatal hypoxia. J. Dev. Orig. health Dis. 4, 328–337. issn 2040-1752. Available from. 10.1017/S204017441300010X 24970726

[B29] Gkousioudi A. Yu X. Ferruzzi J. Qian J. Wainford R. D. Seta F. (2022). Biomechanical properties of mouse carotid arteries with diet-induced metabolic syndrome and aging. Front. Bioeng. Biotechnol. 10, 862996. issn 22964185. Available from. 10.3389/FBIOE.2022.862996 35392404 PMC8980683

[B30] Gokcen T. Inci K. Inci E. E. Sevgen O. Serdar U. (2022). Allopurinol treatment reduced vascular remodeling and improved vascular functions in monocrotaline-induced pulmonary hypertensive rats. Pulm. Pharmacol. Ther. 77, 102166. issn 15229629. Available from. 10.1016/j.pupt.2022.102166 36165827

[B31] Gonzalez-Candia A. Veliz M. Carrasco-Pozo C. Castillo R. L. Cárdenas J. C. Ebensperger G. (2019). Antenatal melatonin modulates an enhanced antioxidant/pro-oxidant ratio in pulmonary hypertensive newborn sheep. Redox Biol. 22, 101128. Available from. 10.1016/j.redox.2019.101128 30771751 PMC6375064

[B32] Greenwald S. E. Moore J. E. Jr. Rachev A. Kane T. P. C. Meister J. J. (1997). Experimental investigation of the distribution of residual strains in the artery wall. J. Biomechanical Eng. 119, 438–444. 10.1115/1.2798291 9407283

[B33] Gundiah N. Ratcliffe M. B. Pruitt L. A. (2009). The biomechanics of arterial elastin. J. Mech. Behav. Biomed. Mater. 2, 288–296. issn 17516161. Available from. 10.1016/j.jmbbm.2008.10.007 19627833

[B34] Guo X. Gong C. Zhai Y. Yu H. Li J. Sun H. (2023). Biomechanical characterization of normal and pathological human ascending aortic tissues via biaxial testing Experiment, constitutive modeling and finite element analysis. Comput. Biol. Med. 166, 107561. Available from. 10.1016/J.COMPBIOMED.2023.107561 37857134

[B35] Haskett D. Johnson G. Zhou A. Utzinger U. Vande Geest J. (2010). Microstructural and biomechanical alterations of the human aorta as a function of age and location. Biomechanics Model. Mechanobiol. 9, 725–736. 10.1007/S10237-010-0209-7 20354753

[B36] Herrera E. A. Riquelme R. A. Ebensperger G. Reyes R. V. Ulloa C. E. Cabello G. (2010). Long-term exposure to high-altitude chronic hypoxia during gestation induces neonatal pulmonary hypertension at sea level. Am. J. Physiol. Regul. Integr. Comp. Physiol. 299, 1676–1684. Available from. 10.1152/ajpregu.00123.2010 PMC3007194 20881096

[B37] Hirata K. Yaginuma T. O’Rourke M. F. Kawakami M. (2006). Age-related changes in carotid artery flow and pressure pulses: possible implications for cerebral microvascular disease. Stroke 37, 2552–2556. issn 1524-4628. Available from. 10.1161/01.STR.0000242289.20381.F4 16946148

[B38] Holzapfel G. A. Ogden R. W. (2018). Biomechanical relevance of the microstructure in artery walls with a focus on passive and active components. Am. J. physiology. Heart circulatory physiology 315, H540–H549. 10.1152/AJPHEART.00117.2018 29799274

[B39] Huh U. Lee C. W. You J. H. Song C. H. Lee C. S. Ryu D. M. (2019). Determination of the material parameters in the holzapfel-gasser-ogden constitutive model for simulation of age-dependent material nonlinear behavior for aortic wall tissue under uniaxial tension. Appl. Sci. 9, 2851. 10.3390/APP9142851

[B40] Humphrey J. D. (2021). Constrained mixture models of soft tissue growth and remodeling – twenty years after. J. Elast. 145, 49–75. issn 15732681. Available from. 10.1007/S10659-020-09809-1 34483462 PMC8415366

[B41] Hung M. W. Yeung H. Lau C. Poon A. Tipoe G. Fung M. (2017). Melatonin attenuates pulmonary hypertension in chronically hypoxic rats. Int. J. Mol. Sci. 18, 1125. 10.3390/IJMS18061125 28538666 PMC5485949

[B42] Hussain A. Bennett R. T. Tahir Z. Isaac E. Chaudhry M. A. Qadri S. S. (2019). Differential effects of atrial and brain natriuretic peptides on human pulmonary artery: an *in vitro* study. World J. Cardiol. 11, 236–243. issn 1949-8462. Available from. 10.4330/WJC.V11.I10.236 31754411 PMC6859300

[B43] Hutter D. Kingdom J. Jaeggi E. (2010). Causes and mechanisms of intrauterine hypoxia and its impact on the fetal cardiovascular system: a review. Int. J. Pediatr. 2010, 1–9. 10.1155/2010/401323 PMC2963133 20981293

[B44] Kamenskiy A. V. Pipinos I. I. Carson J. S. MacTaggart J. N. Baxter B. T. (2015). Age and disease-related geometric and structural remodeling of the carotid artery. J. Vasc. Surg. 62, 1521–1528. issn 0741-5214. Available from. 10.1016/J.JVS.2014.10.041 25499709

[B45] Kawagoe Y. Green L. White S. Richardson B. (1999). Intermittent umbilical cord occlusion in the ovine fetus near term: effects on behavioral state activity. Am. J. obstetrics Gynecol. 181, 1520–1529. issn 0002-9378. Available from. 10.1016/S0002-9378(99)70399-6 10601938

[B46] Kim Su A. Park S. M. Kim M. N. Kim Y. H. Cho D. H. Ahn C. M. (2012). The relationship between mechanical properties of carotid artery and coronary artery disease. Eur. J. Echocardiogr. 13, 568–573. 10.1093/EJECHOCARD/JER259 22127628

[B47] Klemetti M. M. Teramo K. Kautiainen H. Wasenius N. Eriksson J. G. Laine M. K. (2021). Late-pregnancy fetal hypoxia is associated with altered glucose metabolism and adiposity in young adult offspring of women with type 1 diabetes. Front. Endocrinol. 12, 1. issn 16642392. Available from. 10.3389/fendo.2021.738570 PMC8578885 34777246

[B48] Kochová P. Kuncová J. Švíglerová J. Cimrman R. Miklíková M. Liška V. (2012). The contribution of vascular smooth muscle, elastin and collagen on the passive mechanics of porcine carotid arteries. Physiol. Meas. 33, 1335–1351. issn 1361-6579. Available from. 10.1088/0967-3334/33/8/1335 22813960

[B49] Kucukbas G. N. Doğan Y. (2023). Evaluation of carotid artery Doppler measurements in late-onset fetal growth restriction: a cross-sectional study. J. Surg. Med. 7, 673–677. issn 2602-2079. Available from. 10.28982/JOSAM.7953

[B50] Lanne T. Hansen F. Mangell P. Sonesson B. (1994). Differences in mechanical properties of the common carotid artery and abdominal aorta in healthy males. J. Vasc. Surg. 20, 218–225. issn 0741-5214. Available from. 10.1016/0741-5214(94)90009-4 8040945

[B51] Lasheras J. C. (2007). The biomechanics of arterial aneurysms. Annu. Rev. Fluid Mech. 39, 293–319. isbn 0824307399. issn 00664189. Available from. 10.1146/annurev.fluid.39.050905.110128

[B52] Laubrie J. D. Bezmalinovic A. García-Herrera C. M. Celentano D. J. Herrera E. A. Avril S. (2023). Hyperelastic and damage properties of the hypoxic aorta treated with Cinaciguat. J. Biomechanics 147, 111457. issn 18732380. Available from. 10.1016/j.jbiomech.2023.111457 36701962

[B53] Lee C.-W. Huh U. You J.-H. Lee C.-S. Kim K.-H. Song C.-H. (2018). Computational evaluation for age-dependent material nonlinear behavior of aortic wall tissue on abdominal aortic aneurysms. Appl. Sci. 9, 101. 10.3390/APP9010101

[B54] Liu-Shiu-Cheong Patrick S. K. Weir-McCall J. R. Houston J. G. Struthers A. D. (2020). Allopurinol in patients with pulmonary hypertension associated with chronic lung disease. Int. J. Chronic Obstr. Pulm. Dis. 15, 2015–2024. issn 11782005. Available from. 10.2147/COPD.S260917 PMC7457596 32904701

[B55] Maarman G. J. Lecour S. (2021). Melatonin against pulmonary arterial hypertension: is it ready for testing in patients? Cardiovasc. J. Afr. 32, 57–58. issn 16800745. Available from. 10.5830/CVJA-2021-008 PMC8756062 34143177

[B56] Moghadasi K. Ghayesh M. H. Hu E. Li J. (2024). Nonlinear biomechanics of diseased carotid arteries. Int. J. Eng. Sci. 199, 104070. Available from. 10.1016/J.IJENGSCI.2024.104070

[B57] Mohamed S. F. Khayeka-Wandabwa C. Muthuri S. Ngomi N. Kyobutungi C. Haregu T. (2023). Carotid intima media thickness (CIMT) in adults in the AWI-Gen Nairobi site study: profiles and predictors. Hipertens. Riesgo Vasc. vol. 40, no. 1, pp. 5–15. 10.1016/j.hipert.2022.08.001 36153304 PMC11317065

[B58] Murtada S. I. Kawamura Y. Weiss D. Humphrey J. (2021). Differential biomechanical responses of elastic and muscular arteries to angiotensin II-induced hypertension. J. Biomechanics 119, 110297. issn 18732380. Available from. 10.1016/j.jbiomech.2021.110297 PMC8044024 33647550

[B59] Navarrete A. Chen Z. Aranda P. Poblete D. Utrera A. García-Herrera C. M. (2020). Study of the effect of treatment with atrial natriuretic peptide (ANP) and cinaciguat in chronic hypoxic neonatal lambs on residual strain and microstructure of the arteries. Front. Bioeng. Biotechnol. 8, 590488. issn 22964185. Available from. 10.3389/fbioe.2020.590488 33244466 PMC7683788

[B60] Navarrete A. Inostroza M. Utrera A. Bezmalinovic A. González-Candia A. Rivera E. (2024). Biomechanical effects of hemin and sildenafil treatments on the aortic wall of chronic-hypoxic lambs. Front. Bioeng. Biotechnol. 12, 1406214. 10.3389/FBIOE.2024.1406214 39021365 PMC11252865

[B61] Nelson R. J. Drazen D. L. (2000). Melatonin mediates seasonal changes in immune function. Ann. N. Y. Acad. Sci. 917, 404–415. issn 00778923. Available from. 10.1111/J.1749-6632.2000.TB05405.X 11268368

[B62] Olcese J. M. (2020). Melatonin and female reproduction: an expanding universe. Front. Endocrinol. 11, 85. issn 16642392. Available from. 10.3389/fendo.2020.00085 PMC7067698 32210911

[B63] Olver T. D. Klakotskaia D. Ferguson B. S. Hiemstra J. A. Schachtman T. R. Laughlin M. H. (2016). Carotid artery vascular mechanics serve as biomarkers of cognitive dysfunction in aortic-banded miniature swine that can Be treated with an exercise intervention. J. Am. Heart Assoc. 5 (issn), 2047–9980. Available from. 10.1161/JAHA.116.003248 PMC4889197 27207966

[B64] Pagoulatou S. Z. Ferraro M. Trachet B. Bikia V. Rovas G. Crowe L. A. (2021). The effect of the elongation of the proximal aorta on the estimation of the aortic wall distensibility. Biomechanics Model. Mechanobiol. 20, 107–119. issn 16177940. Available from. 10.1007/s10237-020-01371-y PMC7892736 32737630

[B65] Pandi-Perumal S. R. Trakht I. Srinivasan V. Spence D. Maestroni G. Zisapel N. (2008). Physiological effects of melatonin: role of melatonin receptors and signal transduction pathways. Prog. Neurobiol. 85, 335–353. issn 0301-0082. Available from. 10.1016/J.PNEUROBIO.2008.04.001 18571301

[B66] Papamatheakis D. G. Blood A. Kim J. Wilson S. (2013). Antenatal hypoxia and pulmonary vascular function and remodeling. Curr. Vasc. Pharmacol. 11, 616–640. issn 15701611. Available from. 10.2174/1570161111311050006 24063380 PMC4527655

[B67] Parraguez V. H. Atlagich M. Díaz R. Bruzzone M. E. Behn C. Raggi L. A. (2005). Effect of hypobaric hypoxia on lamb intrauterine growth: comparison between high- and low-altitude native ewes. Reproduction, Fertil. Dev. 17, 497–505. issn 1448-5990. Available from. 10.1071/RD04060 15907274

[B68] Patterson A. J. Zhang L. (2010). Hypoxia and fetal heart development. Curr. Mol. Med. 10, 653–666. issn 15665240. Available from. 10.2174/156652410792630643 20712587 PMC3075953

[B69] Paz A. A. Arenas G. A. Castillo-Galán S. Peñaloza E. Cáceres-Rojas G. Suazo J. (2019). Premature vascular aging in Guinea pigs affected by fetal growth restriction. Int. J. Mol. Sci. 20, 3474. issn 14220067. Available from. 10.3390/IJMS20143474 31311132 PMC6678381

[B70] Peace A. Van Mil A. Jones H. Thijssen D. H. (2018). Similarities and differences between carotid artery and coronary artery function. Curr. Cardiol. Rev. 14, 254–263. issn 1573403X. Available from. 10.2174/1573403X14666180910125638 30198437 PMC6300794

[B71] Pukaluk A. Sommer G. Holzapfel G. A. (2024). Multimodal experimental studies of the passive mechanical behavior of human aortas: current approaches and future directions. Acta Biomater. 178, 1–12. issn 1742-7061. Available from. 10.1016/J.ACTBIO.2024.02.026 38401775

[B72] Ream M. Ray A. M. Chandra R. Chikaraishi D. M. (2008). Early fetal hypoxia leads to growth restriction and myocardial thinning. Am. J. Physiology - Regul. Integr. Comp. Physiology 295, R583–R595. issn 03636119. Available from. 10.1152/AJPREGU.00771.2007 PMC2519936 18509101

[B73] Rivera E. Canales C. Pacheco M. García-Herrera C. Macías D. Celentano D. J. (2021). Biomechanical characterization of the passive response of the thoracic aorta in chronic hypoxic newborn lambs using an evolutionary strategy. Sci. Rep. 11 (1), 13875–13911. isbn 0123456789. issn 20452322. Available from. 10.1038/s41598-021-93267-9 34230509 PMC8260639

[B74] Rivera E. García-Herrera C. González-Candia A. Celentano D. J. Herrera E. A. (2020). Effects of melatonin on the passive mechanical response of arteries in chronic hypoxic newborn lambs. J. Mech. Behav. Biomed. Mater. 112, 104013. January. issn 18780180. Available from. 10.1016/j.jmbbm.2020.104013 32846285

[B75] Rueda-Clausen C. F. Morton J. S. Davidge S. T. (2009). Effects of hypoxia-induced intrauterine growth restriction on cardiopulmonary structure and function during adulthood. Cardiovasc. Res. 81, 713–722. issn 0008-6363. Available from. 10.1093/CVR/CVN341 19088083

[B76] Saw S. N. Tay J. J. H. Poh Y. W. Yang L. Tan W. C. Tan L. K. (2018). Altered placental chorionic arterial biomechanical properties during intrauterine growth restriction. Sci. Rep. 8, 16526–16612. issn 2045-2322. Available from. 10.1038/s41598-018-34834-5 30409992 PMC6224524

[B77] Schneider C. A. Rasband W. S. Eliceiri K. W. (2012). NIH Image to ImageJ: 25 years of image analysis. Nat. Methods 9, 671–675. Available from. 10.1038/nmeth.2089 22930834 PMC5554542

[B78] Sehgal A. Murthi P. Dahlstrom J. E. (2019). Vascular changes in fetal growth restriction: clinical relevance and future therapeutics. J. perinatology official J. Calif. Perinat. Assoc. 39, 366–374. issn 1476-5543. Available from. 10.1038/S41372-018-0287-4 30518801

[B79] Sethi D. Gofur E. M. Munakomi S. (2023). Anatomy, head and neck: carotid arteries. StatPearls. Available online at: https://www.ncbi.nlm.nih.gov/books/NBK545238/. 31424822

[B80] Sherman S. R. Lefferts W. K. Lefferts E. C. Grigoriadis G. Lima N. S. Fernhall B. (2022). The effect of aging on carotid artery wall mechanics during maximal resistance exercise. Eur. J. Appl. Physiology 122, 2477–2488. issn 14396327. Available from. 10.1007/S00421-022-05016-Z 36008691

[B81] Sigaeva T. Destrade M. Martino D. I. Elena S. (2019). Multi-sector approximation method for arteries: the residual stresses of circumferential rings with non-trivial openings. J. R. Soc. Interface 16 (156), 20190023. isbn 0000000167. issn 17425662. Available from. 10.1098/rsif.2019.0023 31337302 PMC6685029

[B82] Skilton M. R. Boussel L. Bonnet F. Bernard S. Douek P. C. Moulin P. (2011). Carotid intima–media and adventitial thickening: comparison of new and established ultrasound and magnetic resonance imaging techniques. Atherosclerosis 215 (2), 405–410. issn 0021-9150. Available from. 10.1016/j.atherosclerosis.2010.12.036 21300355

[B83] Steinhorn R. H. (2017). Persistent pulmonary hypertension of the newborn. Fetal Neonatal Brain Inj., 566–582. Available from. 10.1017/9781316275498.037

[B84] Tong J. Xin Y. F. Zhang Z. Xu X. Li T. (2023). Effect of hypertension on the delamination and tensile strength of ascending thoracic aortic aneurysm with a focus on right lateral region. J. Biomechanics 154, 111615. issn 18732380. Available from. 10.1016/j.jbiomech.2023.111615 37178496

[B85] Torres F. González‐Candia A. Montt C. Ebensperger G. Chubretovic M. Serón‐Ferré M. (2015). Melatonin reduces oxidative stress and improves vascular function in pulmonary hypertensive newborn sheep. J. Pineal Res. 58, 362–373. issn 1600079X. Available from. 10.1111/JPI.12222 25736256

[B86] Walsh M. T. Cunnane E. Mulvihill J. Akyildiz A. Gijsen F. Holzapfel G. (2014). Uniaxial tensile testing approaches for characterisation of atherosclerotic plaques. J. Biomechanics 47, 793–804. issn 18732380. Available from. 10.1016/j.jbiomech.2014.01.017 24508324

[B87] Werner F. Kojonazarov B. Gaßner B. Abeßer M. Schuh K. Völker K. (2016). Endothelial actions of atrial natriuretic peptide prevent pulmonary hypertension in mice. Basic Res. Cardiol. 111, 22–16. issn 14351803. Available from. 10.1007/S00395-016-0541-X 26909880 PMC4766231

[B88] Wiedemann R. Ghofrani H. Weissmann N. Schermuly R. Quanz K. Grimminger F. (2001). Atrial natriuretic peptide in severe primary and nonprimary pulmonary hypertension: response to iloprost inhalation. J. Am. Coll. Cardiol. 38, 1130–1136. issn 07351097. Available from. 10.1016/S0735-1097(01)01490-5 11583893

[B89] Xu X. Liu X. Ma S. Xu Y. Xu Y. Guo X. (2018). Association of melatonin production with seasonal changes, low temperature, and immuno-responses in hamsters. Mol. A J. Synthetic Chem. Nat. Prod. Chem. 23, 703. issn 14203049. Available from. 10.3390/MOLECULES23030703 PMC6017911 29558391

[B90] Yildiz M. Balcioğlu S. A. (2024). Current position and future perspectives of melatonin and its supplements in pulmonary hypertension. Koşuyolu Heart J. 27, 37–39. 10.51645/KHJ.2024.429

[B91] Yosibash Z. Priel E. (2011). P-FEMs for hyperelastic anisotropic nearly incompressible materials under finite deformations with applications to arteries simulation. Int. J. Numer. METHODS Eng. Int. J. Numer. Meth. Engng. 88, 1152–1174. Available from. 10.1002/nme.3213

[B92] Zahn A. Balzani D. (2018). A combined growth and remodeling framework for the approximation of residual stresses in arterial walls. ZAMM Z. fur Angew. Math. Mech. 98, 2072–2100. issn 15214001. Available from. 10.1002/ZAMM.201700273

[B93] Zisapel N. (2018). New perspectives on the role of melatonin in human sleep, circadian rhythms and their regulation. Br. J. Pharmacol. 175, 3190–3199. issn 14765381. Available from. 10.1111/BPH.14116 29318587 PMC6057895

